# EPO-modified bone marrow MSCs alleviate asthma inflammation through enhanced mitochondrial activation and transfer by upregulating HO-1

**DOI:** 10.1186/s10020-025-01371-7

**Published:** 2025-09-29

**Authors:** Yao Zhang, Li-Zhan Chen, Hai-Feng Ou-Yang

**Affiliations:** Department of Respiratory Medicine, Xi’an International Medical Center Hospital, No. 777 Xitai Road, Xi’an, 710101 China

**Keywords:** Asthma, EPO-BM-MSCs, Mitochondrial transfer, TNT, Epithelial cells

## Abstract

**Background:**

Bone marrow mesenchymal stem cells (BM-MSCs) can rejuvenate injured cells through mitochondrial transfer. Our previous study has highlighted the ability of erythropoietin (EPO)-modified BM-MSCs (EPO-BM-MSCs) to relieve asthmatic inflammation. Here, we elucidated whether EPO-BM-MSCs improve asthmatic phenotype by mitochondrial transfer and investigated the underlying mechanism.

**Methods:**

EPO-BM-MSCs and different modified EPO-BM-MSCs were generated. Ovalbumin (OVA)-induced asthma mouse models were established, and mtCC1-2 cells were treated with CoCl_2_ to mimic in vitro asthmatic phenotype. EPO-BM-MSC engraftment and mitochondrial transfer from EPO-BM-MSCs to epithelial cells were assessed by fluorescent microscopy and flow cytometry. Mitochondrial membrane potential, ROS production and tunnelling nanotube (TNT) formation were detected by flow cytometry or fluorescent microscopy.

**Results:**

Intratracheal transplantation of EPO-BM-MSCs alleviated airway inflammation, asthmatic phenotype, and mitochondrial dysfunction in the lungs of OVA-induced asthma mice. Moreover, EPO-BM-MSCs had more efficient effects than BM-MSCs. EPO-BM-MSCs diminished CoCl_2_-triggered mitochondrial dysfunction in mtCC1-2 cells in vitro, which could be reversed by the inhibitors of TNT formation. When CoCl_2_-stimulated mtCC1-2 cells were co-cultured with EPO-BM-MSCs, TNT formation significantly increased. EPO-BM-MSCs were validated to donate mitochondria to mtCC1-2 cells through intercellular TNTs in vitro and pulmonary epithelial cells in vivo. EPO-BM-MSCs-upregulated HO-1 contributed to enhanced mitochondrial transfer and improved anti-inflammatory efficacy. Additionally, M-sec promoted intercellular TNT formation and Miro1 enhanced mitochondrial transfer from EPO-BM-MSCs to mtCC1-2 cells.

**Conclusion:**

Our findings demonstrate that EPO-BM-MSCs rescue epithelial cell injury by mitochondrial donation by upregulating HO-1 to alleviate asthma inflammation, providing novel evidence for the therapeutic potential of EPO-BM-MSCs in asthma.

## Introduction

Asthma constitutes a chronic inflammatory disorder of the airways, intricately involving multiple cellular components. Characterized by heightened airway reactivity, it leads to recurrent symptoms, such as wheezing, shortness of breath, chest tightness, and cough (Agache et al. 2021). The underlying pathogenesis of asthma is multifaceted, encompassing genetics, environment, neuromodulation, and immune dysregulation (Miller et al. 2021). In recent years, mitochondrial dysfunction leading to airway epithelial cell injury and inflammation has been recognized as a significant contributor to the development of asthma (Larson-Casey et al. 2020; Zhao et al. 2023). Current treatment relies on bronchodilators and anti-inflammatory agents, yet asthma control remains challenging (Miller et al. 2021). This underscores the pressing need to delve into and develop novel therapeutic approaches. 

Bone marrow-derived mesenchymal stem cells (BM-MSabbreCs) possess the capability for self-renewal and multi-directional differentiation, enabling them to transform into diverse interstitial tissues, such as cartilage, bone, adipose tissue, and bone marrow hematopoietic tissues (Purwaningrum et al. 2021). In the clinical setting, BM-MSCs have demonstrated their utility in addressing a wide array of intractable diseases, including autoimmune disorders and diabetes (Arshad et al. 2023; Izadi et al. 2022). Emerging evidence has shown the potential efficacy of BM-MSCs in asthma treatment owing to their potent anti-inflammatory and immunomodulatory properties. A crucial example is that BM-MSCs strongly attenuate T helper 2 (Th2) cytokine levels and airway inflammation and hyper-responsiveness in ovalbumin (OVA)-challenged asthma mice (Choi et al. 2022). Furthermore, MSCs by intratracheal administration or tail vein injection can engraft into pulmonary epithelium in OVA-induced asthma mice (Yao et al. 2018; Han et al. 2018). The resident MSCs in the lungs is capable of diminishing OVA-evoked epithelial mitochondrial dysfunction and thus improves allergic airway inflammation in OVA asthma mice (Yao et al. 2018). These findings suggest that airway epithelial cells, serving as the initial phase in the development of asthma, are important target cells for MSCs in modulating the asthmatic phenotype of mice.

Mitochondria, one of the most pivotal organelles within eukaryotic cells, are indispensable for cell biological functions. Intercellular mitochondrial transfer points to the process by which mitochondria migrate between distinct cells, which has garnered considerable attention in disease research (Borcherding and Brestoff 2023; Liu et al. 2023). Intercellular tunnelling nanotubes (TNTs), characterized by their elongated formations stemming from cellular membrane extensions, mediates mitochondria transfer (Lin et al. 2024). Extensive investigations have illuminated that BM-MSCs possess the capability to rescue the function of recipient cells via TNT-mediated mitochondrial donation, thereby exerting therapeutic efficacy in various human diseases (Yao et al. 2023; Sagar et al. 2023). In addition, MSCs show the ability to donate mitochondria to epithelial cells to alleviate epithelial mitochondrial dysfunction in OVA-challenged asthma mice (Yao et al. 2018).

Erythropoietin (EPO) possesses various biological properties, including anti-inflammation, anti-oxidant, angiogenic and immuno-regulatory effects (Sadanandan et al. 2023; Scholz et al. 2019). Our previous study has successfully established EPO-modified BM-MSCs (EPO-BM-MSCs), demonstrating the ability of EPO-BM-MSCs to relieve asthmatic airway inflammation and remodeling (Han et al. 2018). However, the mechanism underlying the protective role of EPO-BM-MSCs in asthmatic phenotype remains largely unclear. In this study, we used in vivo and in vitro asthma models to elucidate the effect of EPO-BM-MSCs on epithelial mitochondrial dysfunction. We further investigated mitochondrial donation from EPO-BM-MSCs to pulmonary epithelial cells and the mechanism governing it. 

## Materials and methods

### BM-MSCs and establishment of EPO-BM-MSCs, GFP-EPO-BM-MSCs, and mGFP-EPO-BM-MSCs

Mouse BM-MSCs (#CP-M131, Procell, Wuhan, China) were used in passage 6–10 in the current study, which were reproduced at 37 °C in a humidified environment with 5% CO_2_using Procell-developed standard medium. As our previous work (Han et al. 2018), EPO-BM-MSCs were generated by infecting BM-MSCs with lentiviral particles expressing EPO coding sequences in media containing polybrene (2 µg/mL). To produce GFP-tagged EPO-BM-MSCs (GFP-EPO-BM-MSCs), lentiviral particles carrying EPO-GFP sequences were used to infect BM-MSCs. To establish EPO-BM-MSCs with GFP-labeled mitochondria (mGFP-EPO-BM-MSCs), EPO-BM-MSCs were transduced with lentivirus particles expressing mitochondrial targeting green fluorescence protein (pCT-MITO-GFP) from System Biosciences (Palo Alto, CA, USA).

### Animal studies

We conducted all animal procedures under approval from Xi’an International Medical Center Hospital Animal Ethics and Experimentation Committee (Approval No. GJYX-KY-2023-006) using six-eight-week-old female C57BL/6 mice (Vital River Laboratory, Beijing, China, *n* = 78), who were divided into eight groups: control (*n* = 6), OVA (*n* = 6), OVA + BM-MSCs (*n* = 6), OVA + EPO-BM-MSCs (*n* = 6), OVA + EPO-BM-MSCs + SnPPIX (*n* = 6), control + GFP-EPO-BM-MSCs (*n* = 15), OVA + GFP-EPO-BM-MSCs (*n* = 15), and OVA + mGFP-EPO-BM-MSCs (*n*= 18). As our previous study (Ou-Yang et al. 2011), we established OVA-induced asthma mouse models. Briefly, on days 1 and 8, mice underwent intraperitoneal sensitization with 100 µg of OVA (#E6337, Macklin, Shanghai, China), which was adsorbed to 9% aluminum hydroxide hydrate (#A1577, Sigma-Aldrich, Saint-Aubin, France). Subsequently, from days 15 to 17, these mice were intratracheally challenged with 100 µg of OVA dissolved in PBS. In the control + GFP-EPO-BM-MSCs, OVA + GFP-EPO-BM-MSCs and OVA + mGFP-EPO-BM-MSCs groups, modified EPO-BM-MSCs were given by an intratracheal route on day 18 and further procedures were described as below. In the other five groups, BM-MSCs or EPO-BM-MSCs (5 × 10^5^cells/mouse) were intratracheally delivered in 50 µL of PBS at 18–24 h prior to intratracheal challenge of OVA, and each mice group was euthanized at different times as described below. In addition, SnPPIX administration was performed by intraperitoneal injection of SnPPIX (10 mM/kg, Frontier Scientific, Franckfurt, Germany) at the time of MSC intratracheal delivery. In contrast, control mice received PBS only in the same way. Prior to sacrifice, airway pressure-time index (APTI) of mouse lungs was evaluated as reported elsewhere (Ou-Yang et al. 2011). In brief, after being intubated and ventilated, mice were subjected to intravenous injection of acetylcholine (50 µg/kg) following anesthetization. Bronchoalveolar lavage fluid (BALF) and lung tissues were collected from the sacrificial mice as described elsewhere (Han et al. 2018). Cell count in BALF was performed by the Wright-Giemsa staining method (Dhlamini et al. 2022). Briefly, after centrifugalization, the sediment cells were stained and observed under microscopy.

### Histological analysis by hematoxylin and Eosin (H&E) staining

For histopathologic assessment, we carried out H&E staining. Briefly, collected mouse lungs underwent fixation in 4% paraformaldehyde, followed by paraffin embedding. Subsequently, slides (4 μm) of the lungs were subjected to H&E staining with a commercial kit as recommended by the supplier (Solarbio, Beijing, China). Following image capture using the Olympus GX-71 microscope (Olympus, Tokyo, Japan), the histological alterations and inflammation score were analyzed.

Inflammation scores were evaluated by a blinded observer on a scale of 0–4 based on the degree of peribronchiolar inflammatory cell infiltration and tissue damage, where 0 = no inflammation, 1 = mild, 2 = moderate, 3 = severe, and 4 = very severe inflammation.

### Evaluation of EPO-BM-MSC engraftment

The mice in the control + GFP-EPO-BM-MSCs and OVA + GFP-EPO-BM-MSCs groups were used to observe the engraftment of EPO-BM-MSCs in the lungs. At 1 h, 4 h, 8 h, 1 d and 4 d post intratracheal delivery of GFP-EPO-BM-MSCs on day 18, mouse lungs were harvested and checked for MSC engraftment using fluorescent microscopy and flow cytometry analysis (*n* = 3 mice for each time point). For fluorescent microscopy, after being cryosectioned, sections of the lungs were subjected to nuclear staining with 4’,6-diamidino-2-phenylindole (DAPI) (Servicebio, Wuhan, China). Image acquisition was done by the Olympus FV3000 confocal microscope. In flow cytometry assay, single-cell suspensions were first made by digesting lung tissues with collagenase I (Servicebio) and dispase (Beyotime). Following the application of a 70 μm mesh, we quantified the percentage of GFP-positive cells (GFP-EPO-BM-MSCs) using a FACS Calibur flow cytometer equipped with CellQuest software (BD Biosciences, Oxford, UK).

### ELISA for IL-4, IL-5, IL-13, thymic stromal lymphopoietin (TSLP) and IL-33 secretion

The collected mouse BALF was subjected to ELISA for these detections. The commercially available Mouse IL-13 ELISA Kit (Multi Sciences, Hangzhou, China), Mouse IL-5 ELISA Kit (Abcam, Cambridge, UK), Mouse IL-4 ELISA Kit (Beyotime, Shanghai, China), Mouse IL-33 ELISA Kit (Abcam) and Mouse TSLP ELISA Kit (Multi Sciences) were applied in accordance with the protocols suggested by the vendors. For absorbance measurement, an iMark Microplate Reader (Bio-Rad, Glattbrugg, Switzerland) was employed.

Lung tissues were harvested from each group of mice. Tissues were homogenized in lysis buffer (RIPA buffer with protease and phosphatase inhibitors) using a tissue homogenizer. The homogenates were centrifuged at 12,000 × g for 15 min at 4 °C to remove debris. The supernatant was collected, and protein concentration was quantified via BCA assay. Cytokine levels (e.g., IL-4, IL-5, IL-13) in lung tissue homogenates were analyzed using ELISA kits per the manufacturer’s instructions.

###  Constructs and transfection of EPO-BM-MSCs

The specific M-sec-siRNA pools (si-M-sec), Miro1-siRNA pools (si-Miro1) and nontarget siRNA control (si-NC) were designed and generated by MedChemExpress (Shanghai, China). As suggested by the supplier (Thermo Fisher Scientific, Geel, Belgium), si-M-sec, si-Miro1 or si-NC was transient introduced into EPO-BM-MSCs using Lipofectamine 3000 as the transfection reagent.

### mtCC1-2 cell culture and treatment

For all in vitro experiments, we used a mouse-derived Clara cell line mtCC1-2 and propagated the cell Line in 10% FBS DMEM (Bovogen Biologicals, Springvale, Victoria, Australia) enriched with 1% antibiotic (penicillin/streptomycin, Beyotime). To produce an in vitro asthmatic model, mtCC1-2 cells were subjected to stimulation of 400 µM of CoCl_2_ (Sigma-Aldrich, Milano, Italy) for 12 h. To analyze mitochondrial transport from EPO-BM-MSCs to mtCC1-2 cells, CFSE-labeled and CellTrace Violet-labeled mtCC1-2 cells were generated using CFSE (green, Thermo Fisher Scientific) and CellTrace Violet (blue, Thermo Fisher Scientific).

###  Determination of mitochondrial transfer from EPO-BM-MSCs to epithelial cells

For in vivo analysis, the mice in the OVA + mGFP-EPO-BM-MSCs group were sacrificed at different time points (5 min, 30 min, 1 h, 4 h, 8 h, and 24 h) after MSC intratracheal delivery on day 18 and their lungs were harvested, fixed, and subjected to fluorescent microscopy after nuclear staining of DAPI.

For in vitro assays, CFSE-labeled mtCC1-2 cells were co-cultured with MitoTracker^®^ Red (Mito)-labeled EPO-BM-MSCs, or CellTrace Violet-labeled mtCC1-2 cells were co-cultured with untransfected, si- or si-Miro1-transfected mGFP-EPO-BM-MSCs. Co-culture was performed for 24 h in routine media or media containing Gap26 (0.25 mg/mL, APExBIO, Boston, MA, USA) or SnPPIX (10 µM). Analysis of mitochondrial transfer from EPO-BM-MSCs to mtCC1-2 cells was done by flow cytometry analysis and fluorescent microscopy (Olympus FV3000). The percentage of mtCC1-2 cells labeled with GFP and CellTrace Violet was determined by FACS Calibur.

### Detection of mitochondrial membrane potential (MMP) and ROS production

For in vivo analysis of MMP, a commercial Mitochondria Isolation Kit (Sigma-Aldrich) was applied to prepare mitochondria from mouse lungs as per the manufacturer’s instructions. The isolated mitochondria were incubated with JC-1 fluorescence probes (Sigma-Aldrich), and MMP was analyzed by FACS Calibur flow cytometer (BD Biosciences), with results presented as the mean fluorescence intensity in the FL2 channel.

For in vitro MMP analysis, mtCC1-2 cells (untreated, CoCl₂-stimulated, or co-cultured with EPO-BM-MSCs) were stained with JC-1. Flow cytometry gating strategies included: (1) excluding dead cells and debris by forward scatter (FSC)/side scatter (SSC) gating; (2) isolating single cells to eliminate aggregates; (3) defining high membrane potential cells (red fluorescence) and low membrane potential cells (green fluorescence) using untreated cells as negative controls. Data were expressed as the ratio of Red/Green fluorescence intensity.

For in vitro mitochondrial ROS detection, mtCC1-2 cells were incubated with MitoSOX™ Red (Thermo Fisher Scientific) after different treatments. Flow cytometry analysis involved FSC/SSC gating to exclude debris, followed by gating on single cells. ROS-positive cells were identified by FL2 channel fluorescence intensity, with the threshold set using unstimulated control cells. The mean fluorescence intensity in the FL2 channel was recorded using FACS Calibur.

### Microscopic analysis of TNT formation

To assess TNT formation, we labeled mitochondria with Mito (showing a red fluorescence) and formed TNTs with Actin-Trasker Green (F-actin, showing a green fluorescence) after cell culture or co-culture. mtCC1-2 cells, CoCl_2_-stimulated mtCC1-2 cells, EPO-BM-MSCs, mtCC1-2 cells co-cultured with EPO-BM-MSCs, CoCl_2_-stimulated mtCC1-2 cells co-cultured with EPO-BM-MSCs, si-NC-transfected EPO-BM-MSCs, or si-M-sec-transfected EPO-BM-MSCs were observed by fluorescent microscopy (Olympus FV3000). The percentage of TNT-forming cells was determined using ImageJ (National Institutes of Health, Bethesda, MD, USA).

### Immunoblotting

For protein extraction from lung samples or cultured cells, the commercially available Protein Extraction Kit was used as described by the supplier (Beyotime). After quantification by BCA assay (Thermo Fisher Scientific), protein (25 µg/lane) was gel-electrophoresed on 10% SDS polyacrylamide gels. The resulting gels were electroblotted to PVDF membranes (Beyotime), followed by probing with antibodies including anti-cleaved caspase 9 (rabbit pAb, #PA5-105271, Invitrogen, Saint-Aubin, France, 1 to 1,500), anti-caspase 9 (rabbit pAb, #10380-1-AP, Proteintech, Wuhan, China, 1 to 800), anti-cleaved caspase 3 (rabbit pAb, #PA5-114687, Invitrogen, 1 to 1,000), anti-caspase 3 (mouse mAb, #43-7800, Invitrogen, 1 to 500), anti-HO-1 (rabbit mAb, #ab189491, Abcam, 1 to 2,000), anti-M-sec (rabbit pAb, #ab196659, Abcam, 1 to 2,500), anti-Miro1 (mouse mAb, #ab188029, Abcam, 1 to 1,000), or anti-GAPDH (mouse mAb, #60004-1-Ig, Proteintech, 1 to 50,000). For blot quantification, the LAS-4000 Mini Image Analyzer (Fuji Film, Minamiashigara, Japan) was employed.

### Quantitative real-time PCR

Total RNA was isolated from lung tissues using the AxyPrep Multisource Total RNA kit (AXYGEN, USA). Subsequently, this RNA was reverse transcribed into cDNA using a first strand cDNA synthesis kit (Novoprotein, China). Gene transcripts were then quantified using Novostart SYBR qPCR SuperMix Plus (Novoprotein, China) on a 7300 Plus Real-Time PCR System (Applied Biosystems, USA), following the manufacturer’s instructions. The sequences of primers were used as follows: mouse IL-4 forward: 5’-GGTCTCAACCCCCAGCTAGT-3’, reverse: 5’-GCCGATGATCTCTCTCAAGTGAT-3’; mouse IL-5 forward: 5’-GCAATGAGACGATGAGGCTTC-3’, reverse: 5’-GCCCCTGAAAGATTTCTCCAATG − 3’; mouse GAPDH forward: 5’- AGGTCGGTGTGAACGGATTTG-3’, reverse: 5’-CGCCACGAGCAGGAATGAGAAG-3’. The relative mRNA level was calculated by 2^−ΔΔCt^ method.

### Statistical analysis

All experiments were repeated using at least three biological repeats and presented as mean ± SD. Significance analyses were performed using one-way or two-way ANOVA or unpaired *t*-test. A *p* value less than 0.05 indicated statistical significance. *P < 0.05, **P < 0.01, ***P < 0.001.

## Results

### Intratracheal transplantation of EPO-BM-MSCs results in alleviative airway inflammation in OVA-induced asthma mice

**Fig. 1:**
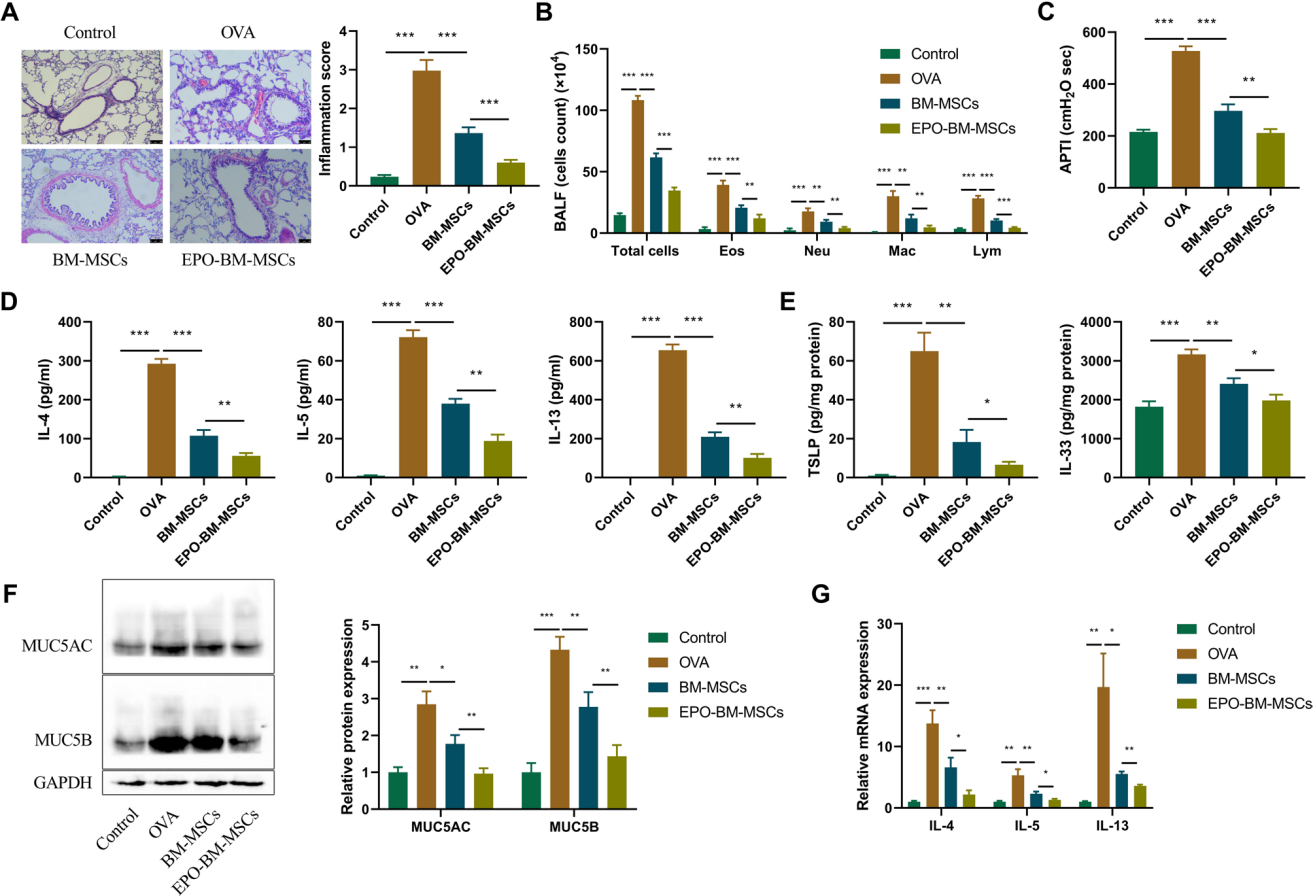
EPO-BM-MSCs relieve airway inflammation in asthma mice induced by OVA. An OVA-induced mouse model of asthma was established, which was accompanied with or without intratracheal administration of EPO-BM-MSCs or BM-MSCs. At the end point, the BALF and lung tissues were obtained. Each group included six mice. **A** H&E staining of lung sections for histopathological observation. Scale bars: 50 μm. **B** Total cells, eosinophils (Eos), neutrophils (Neu), macrophages (Mac), and lymphocytes (Lym) were counted in the BALF. **C** Airway pressure-time index (APTI) was used to evaluate airway responsiveness. **D** and **E** ELISA for IL-4, IL-5, IL-13, TSLP and IL-33 secretion levels in the BALF. **F** Western blot analysis of MUC5AC and MUC5B expression in lung tissues. **G** Quantitative PCR analysis of pro-inflammatory cytokines (IL-4, IL-5, IL-13) in lung tissue from OVA-induced asthma mice treated with PBS, BM-MSCs, or EPO-BM-MSCs. *P < 0.05, **P < 0.01 vs. OVA group. *P < 0.05, **P < 0.01, ***P < 0.001

To further dissect the activity of EPO-BM-MSCs in affecting OVA-induced asthmatic phenotype, we established an OVA-induced mouse model of asthma and performed EPO-BM-MSCs or BM-MSCs local transplantation via intratracheal administration. Histopathological observation by H&E staining revealed the enhanced peribronchiolar infiltration of inflammatory cells and elevated inflammation score in lungs of OVA-induced mice (Fig. 1A). By analyzing cell count in BALF, we found that OVA-induced mice displayed increased total cells, eosinophils (Eos), neutrophils (Neu), macrophages (Mac), and lymphocytes (Lym) in their BALF compared with control mice (Fig. 1B). Meantime, OVA-induced mice had higher airway responsiveness, as evidenced by the enhanced airway pressure-time index (APTI) (Fig. 1C). In addition, OVA-induced mice exhibited higher levels of Th2 cytokines (IL-4, IL-5 and IL-13) and epithelium-derived cytokines (TSLP and IL-33) in their BALF than control mice (Fig. 1D and E). Together, these data confirm the successful establishment of the OVA-induced mouse model of asthma.

Compared with the OVA model group, BM-MSCs administration significantly decreased inflammation score in mouse lungs (Fig. 1A), reduced total cell count and inflammatory cell count in BALF (Fig. 1B), as well as counterbalanced airway hyperreactivity (Fig. 1C). Similarly, BM-MSCs could lead to a striking downregulation in IL-4, IL-5, IL-13, TSLP and IL-33 levels in BALF of OVA-induced mice (Fig. 1D and E). These results validate the anti-inflammation efficacy of BM-MSCs in asthma mice. Intriguingly, when compared to the BM-MSCs group, EPO-BM-MSCs were more efficient for diminishing inflammation (Fig. 1A and B), airway responsiveness (Fig. 1C), and inflammatory cytokine secretion (Fig. 1D and E). Therefore, we conclude that EPO-BM-MSCs have strong ability to relieve OVA-induced airway inflammation in asthma mice. Additionally, Western blot analysis of lung tissues revealed that OVA challenge significantly upregulated the expression of mucin markers MUC5AC and MUC5B, while treatment with EPO-BM-MSCs markedly reduced their levels (Fig. 1F).

To further validate the inflammatory cytokine profile, cytokine levels in lung tissue were analyzed. Similar to BALF results, OVA - challenged mice showed significantly higher pro-inflammatory cytokine (IL-4, IL-5, IL-13) levels in lung tissues than the control group (*p* < 0.05). Treatment with BM-MSCs and EPO-BM-MSCs reduced these cytokines, with EPO-BM-MSCs exhibiting a more pronounced inhibitory effect on IL-4, IL-5, and IL-13 in lung tissues than BM-MSCs (*p* < 0.05) (Fig. 1G). These lung tissue results corroborate the BALF findings, strengthening the evidence for the anti-inflammatory effects of EPO-BM-MSCs in asthmatic mice.

### EPO-BM-MSCs act for alleviation of mitochondrial dysfunction in the lungs of OVA-induced asthma mice

**Fig. 2:**
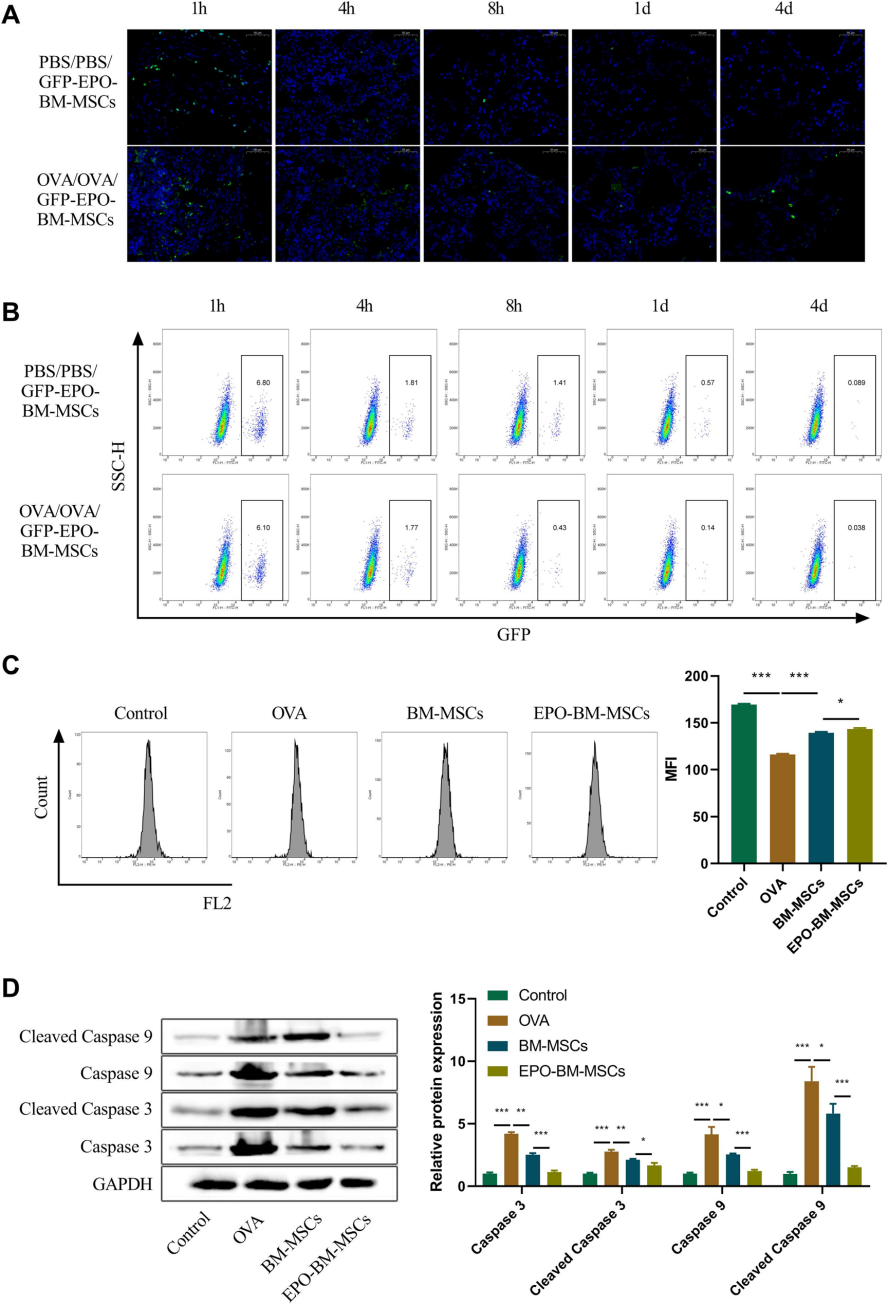
EPO-BM-MSCs ameliorate epithelial mitochondrial function in the lungs of OVA-induced asthma mice. **A** and **B** Fluorescence microscopy (**A**) and flow cytometry analysis (**B**) of lung tissues of PBS-treated mice and OVA-induced asthma mice at various time points (1 h, 4 h, 8 h, 1 d, and 4 d) after intratracheal transplantation of GFP-tagged EPO-BM-MSCs (GFP-EPO-BM-MSCs). Scale bars: 50 μm. **C** Flow cytometry for MMP in lung epithelium of OVA-induced asthma mice accompanied with or without intratracheal administration of EPO-BM-MSCs or BM-MSCs. **D** Immunoblotting of lung tissues of OVA-induced asthma mice accompanied with or without intratracheal administration of EPO-BM-MSCs or BM-MSCs. *P < 0.05, **P < 0.01, ***P < 0.001

In order to examine the distribution and survival of EPO-BM-MSCs following transplantation, we generated GFP-tagged EPO-BM-MSCs (GFP-EPO-BM-MSCs) and used them to treat OVA-induced mice via intratracheal administration. Fluorescence microscopy and flow cytometry analysis of lung tissues were performed at various time points (1 h, 4 h, 8 h, 1 d, and 4 d). After cell transplantation, the distribution and survival of GFP-EPO-BM-MSCs in the PBS group and the OVA group were comparable in lung tissues at 1 and 4 h (Fig. 2A). The number of GFP-EPO-BM-MSCs in mouse lungs time-dependently reduced in both groups (Fig. 2A). Remarkably, GFP-EPO-BM-MSCs in the PBS group disappeared on day 1 after transplantation, while they were still detected in the OVA group (Fig. 2A). More interestingly, in OVA-induced mice, a notable population of GFP-EPO-BM-MSCs was observed on day 4 post transplantation (Fig. 2A), highlighting the higher engraftment of EPO-BM-MSCs into the lungs in OVA-induced mice compared with PBS controls. This finding was also confirmed by flow cytometry of lung tissues (Fig. 2B).

We then analyzed the influence of EPO-BM-MSCs in the activation and apoptosis of epithelial mitochondria in the lungs. Through flow cytometry, we found that OVA-induced mice displayed impaired mitochondrial membrane potential (MMP) compared with control mice (Fig. 2C). BM-MSCs administration enhanced MMP in OVA-induced mice, and EPO-BM-MSCs were more effective when compared with BM-MSCs (Fig. 2C). Subsequent immunoblot analysis of lung homogenates revealed that OVA induction resulted in elevated protein levels of mitochondria apoptosis-related factors (caspase 3, cleaved caspase 3, caspase 9, cleaved caspase 9) in mice (Fig. 2D). Local transplantation of BM-MSCs significantly decreased the expression of the four factors in the lungs of OVA-induced mice, and EPO-BM-MSCs had more efficient effects than BM-MSCs (Fig. 2D). Taken together, these findings indicate that EPO-BM-MSCs can ameliorate epithelial mitochondrial function and suppress apoptosis in the lungs of OVA-induced asthma mice.

### EPO-BM-MSCs diminish CoCl_2_-triggered mitochondrial dysfunction in mtCC1-2 cells in vitro

**Fig. 3:**
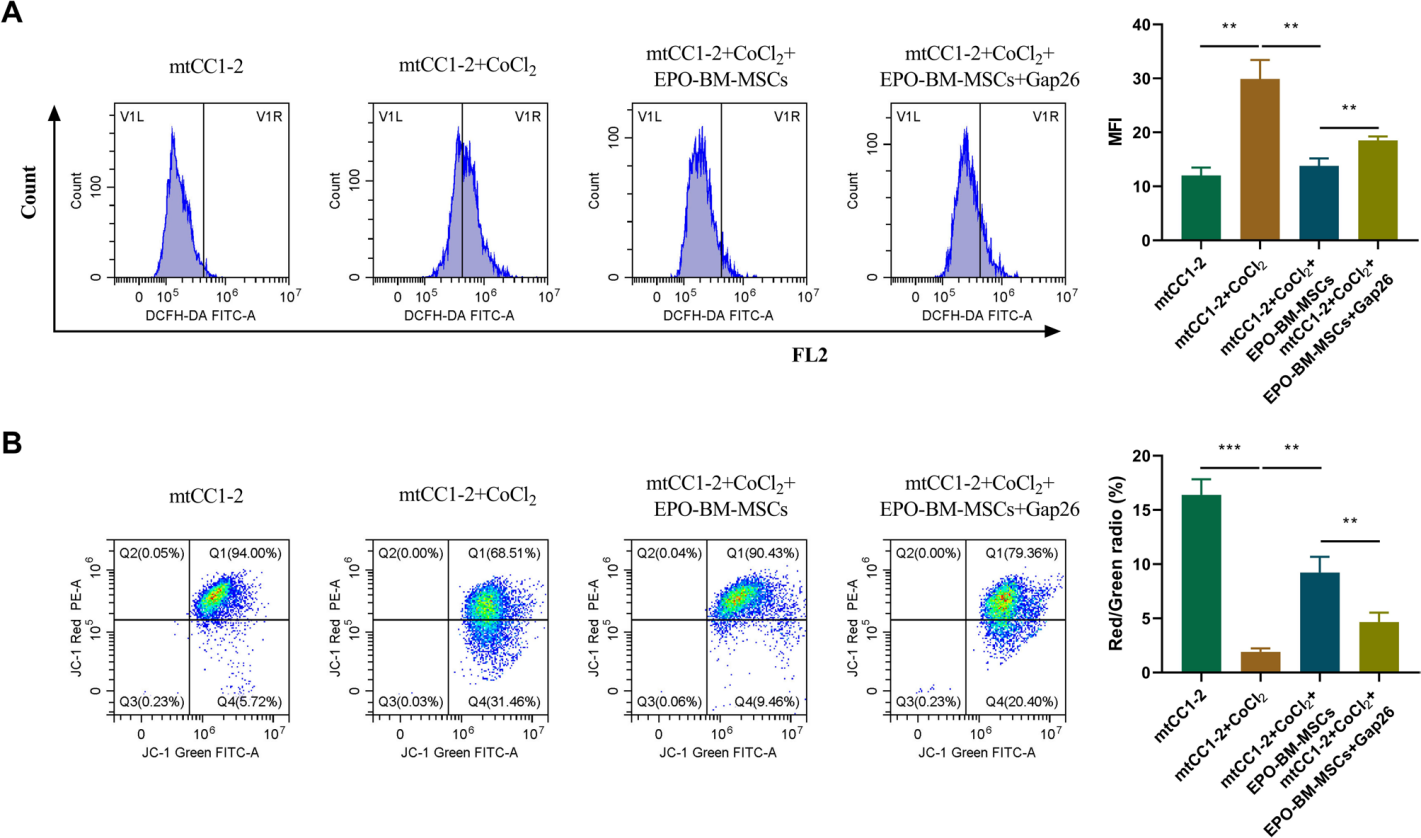
EPO-BM-MSCs mitigate CoCl_2_-triggered mitochondrial dysfunction in mtCC1-2 cells *in vitro*. **A** and **B** Mouse mtCC1-2 cells were stimulated with or without CoCl_2_ for 12 h, or CoCl_2_-stimulated mtCC1-2 cells were co-cultured with EPO-BM-MSCs in normal media or media containing Gap26 for 24 h. **A** ROS content detection by flow cytometry in treated mtCC1-2 cells. **B** MMP of treated mtCC1-2 cells evaluated by flow cytometry. *P < 0.05, **P < 0.01, ***P < 0.001

Next, we further evaluated the protective efficacy of EPO-BM-MSCs on epithelial cell mitochondrial function using a murine lung epithelial cell line mtCC1-2 in vitro. Toward this, we performed co-culture experiments of EPO-BM-MSCs with CoCl_2_-triggered mtCC1-2 cells. Using flow cytometry, we observed that treatment of CoCl_2_ led to a significant elevation in mitochondrial ROS production in mtCC1-2 cells (Fig. 3A). Co-culture with EPO-BM-MSCs markedly decreased mitochondrial ROS content of mtCC1-2 cells under CoCl_2_ (Fig. 3A). Moreover, flow cytometry results showed that treatment of CoCl_2_ strongly impaired MMP of mtCC1-2 cells, while co-culture with EPO-BM-MSCs reversed CoCl_2_-triggered MMP impairment (Fig. 3B). These data show that EPO-BM-MSCs can rescue CoCl_2_-triggered mitochondrial dysfunction in mtCC1-2 cells.

Previous reports have proven that the therapeutic efficacy of MSCs is dependent on their mitochondrial transfer ability through intercellular TNTs (Yao et al. 2023; Sagar et al. 2023). To preliminarily analyze whether the observed effects of EPO-BM-MSCs are due to mitochondrial transfer from MSCs to epithelial cells, the co-culture medium was added with Gap26, which can suppress the formation of intercellular TNTs (Sinclair et al. 2016). Of note, treatment of Gap26 had a counteracting impact on EPO-BM-MSCs-mediated ROS reduction (Fig.3A) and MMP restoration (Fig. 3B) in mtCC1-2 cells, suggesting that the mitigatory function of EPO-BM-MSCs in CoCl_2_-triggered epithelial cell mitochondrial dysfunction may be due to TNT-mediated mitochondrial transfer.

### In vitro and in vivo mitochondrial transfer from EPO-BM-MSCs to epithelial cells

**Fig. 4:**
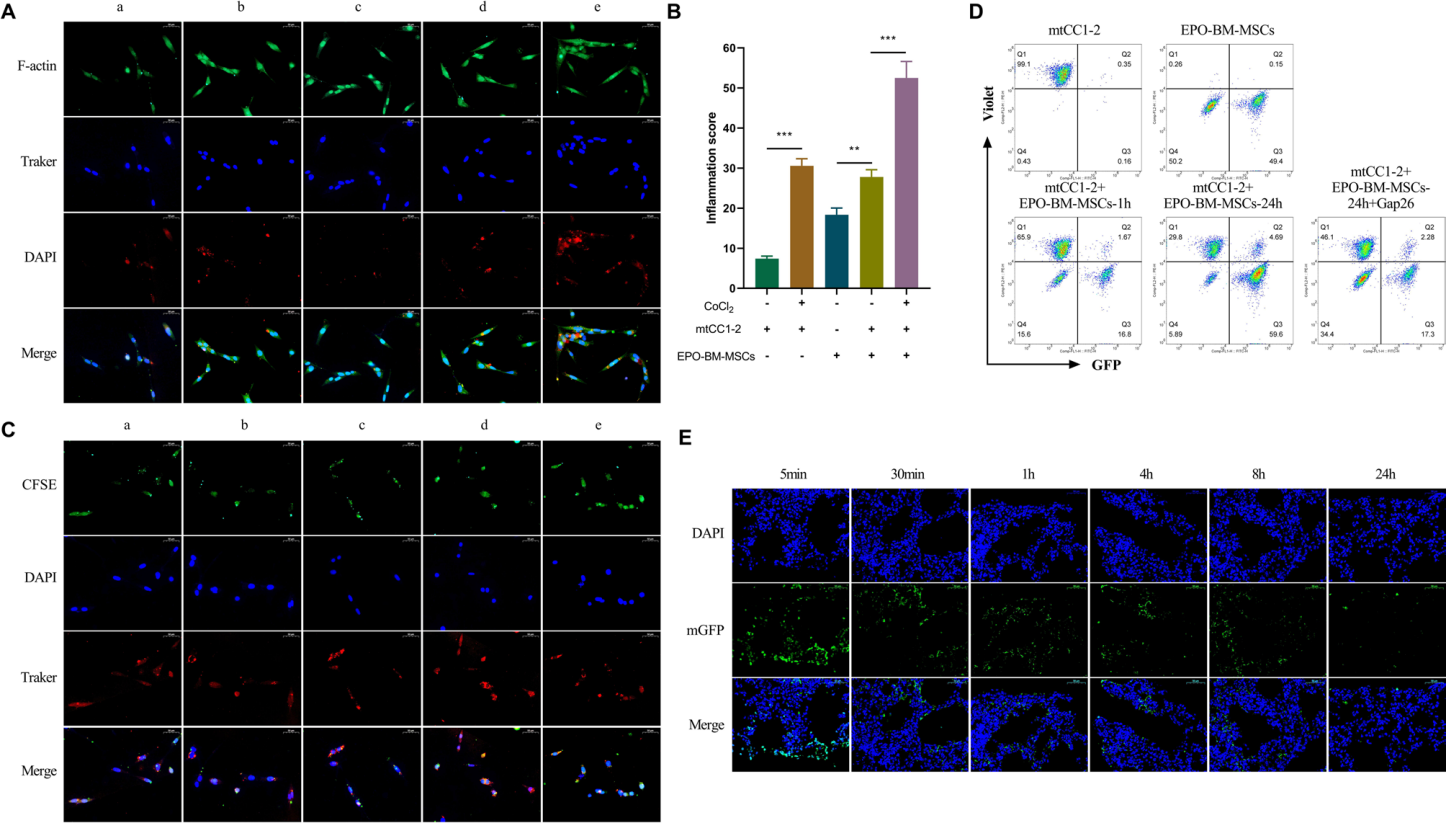
EPO-BM-MSCs donate mitochondria to pulmonary epithelial cells *in vitro* and *in vivo*. **A** and **B** Cellular mitochondria were labeled with MitoTracker Red (Mito, showing a red fluorescence), and formed TNTs were labeled with Actin-Trasker Green (F-actin, showing a green fluorescence). Fluorescence microscopy and the percentage of TNT-forming cells between normal mtCC1-2 cells (a), mtCC1-2 cells under CoCl_2_ treatment (b), EPO-BM-MSCs (c), EPO-BM-MSCs and mtCC1-2 cells (d), and EPO-BM-MSCs and CoCl_2_-stimulated mtCC1-2 cells (e). Scale bars: 50 μm. **C** CFSE-labeled mtCC1-2 cells (showing a green fluorescence) were co-cultured with EPO-BM-MSCs with Mito-labeled mitochondria (showing a red fluorescence) for 24 h, followed by fluorescence microscopy. Scale bars: 100 μm. **D** EPO-BM-MSCs with GFP-mitochondria (mGFP-EPO-BM-MSCs, showing a green fluorescence) were co-cultured with mtCC1-2 cells labeled with CellTrace Violet (showing a blue fluorescence) in normal media or media containing Gap26 for the indicated time point. Flow cytometry analyzed the percentage of mtCC1-2 cells labeled with GFP and CellTrace Violet. **E** OVA-induced asthma mice were performed with intratracheal administration of mGFP-EPO-BM-MSCs. At 5 min, 30 min, 1 h, 4 h, 8 h, and 24 h post transplantation, fluorescence microscopy was used to observe the penetration of mGFP-EPO-BM-MSCs in mouse lungs and the transfer of GFP-labeled mitochondria to pulmonary cells. Scale bars: 50 μm. *P < 0.05, **P < 0.01, ***P < 0.001

To provide further insights into mitochondrial transport from EPO-BM-MSCs to epithelial cells, we first analyzed TNT formation between EPO-BM-MSCs and mtCC1-2 cells. To this end, we labeled mitochondria with MitoTracker Red (Mito, showing a red fluorescence) and formed TNTs with Actin-Trasker Green (F-actin, showing a green fluorescence). Through fluorescence microscopy, we observed the formation of clear TNT-like structures between mtCC1-2 cells under CoCl_2_ treatment, between EPO-BM-MSCs, between EPO-BM-MSCs and mtCC1-2 cells, and between EPO-BM-MSCs and CoCl_2_-stimulated mtCC1-2 cells (Fig. 4A). The evaluation of the percentage of TNT-forming cells was also confirmed the formation status of intercellular TNTs (Fig. 4B). Remarkably, the percentage of TNT-forming cells in the EPO-BM-MSCs and CoCl_2_-stimulated mtCC1-2 group was the largest (Fig. 4B), providing the possibility for mitochondrial transport from EPO-BM-MSCs to epithelial cells.

We next elucidated mitochondrial transport from EPO-BM-MSCs to epithelial cells in vitro and in vivo. CFSE-labeled mtCC1-2 cells (showing a green fluorescence) were co-cultured with EPO-BM-MSCs with Mito-labeled mitochondria (showing a red fluorescence) for 24 h. Using fluorescence microscopy, we observed clear transfer of Mito-labeled mitochondria from EPO-BM-MSCs to CFSE-labeled mtCC1-2 cells at different time points (Fig. 4C). It was worth noting that mitochondrial transfer could be detected as early as 4 h after co-culture, and the number of Mito-labeled mitochondria transferred to mtCC1-2 cells increased over time (Fig. 4C). To avoid MitoTracker leakage, we generated EPO-BM-MSCs with GFP-mitochondria (mGFP, showing a green fluorescence) and co-cultured them with mtCC1-2 cells labeled with CellTrace Violet (showing a blue fluorescence) in normal media or media containing Gap26. Through flow cytometry, we found the increased percentage of GFP-positive mtCC1-2 cells after 24 h of co-culture, while administration of Gap26 could partially abolish the increase (Fig. 4D), demonstrating that intercellular TNTs play crucial roles in mediating mitochondrial transport from EPO-BM-MSCs to mtCC1-2 cells.

The fact of mitochondrial transport from EPO-BM-MSCs to mtCC1-2 cells in vitro led us to further examine if engrafted EPO-BM-MSCs can donate mitochondria to bronchial epithelial cells in OVA-induced asthma mice. At 5 min after cell transplantation, the mGFP-EPO-BM-MSCs penetrated the lungs of OVA-challenged mice (Fig. 4E). GFP-labeled mitochondria could be observed in pulmonary cells after 1 h of transplantation, and their numbers continued to escalate in pulmonary cells over the subsequent time points, evident even at 24 h (Fig. 4E), suggesting that EPO-BM-MSCs are capable of donating mitochondria to pulmonary epithelial cells.

### EPO-BM-MSCs-mediated upregulation of HO-1 leads to enhanced mitochondrial transfer and improved anti-inflammatory efficacy

**Fig. 5:**
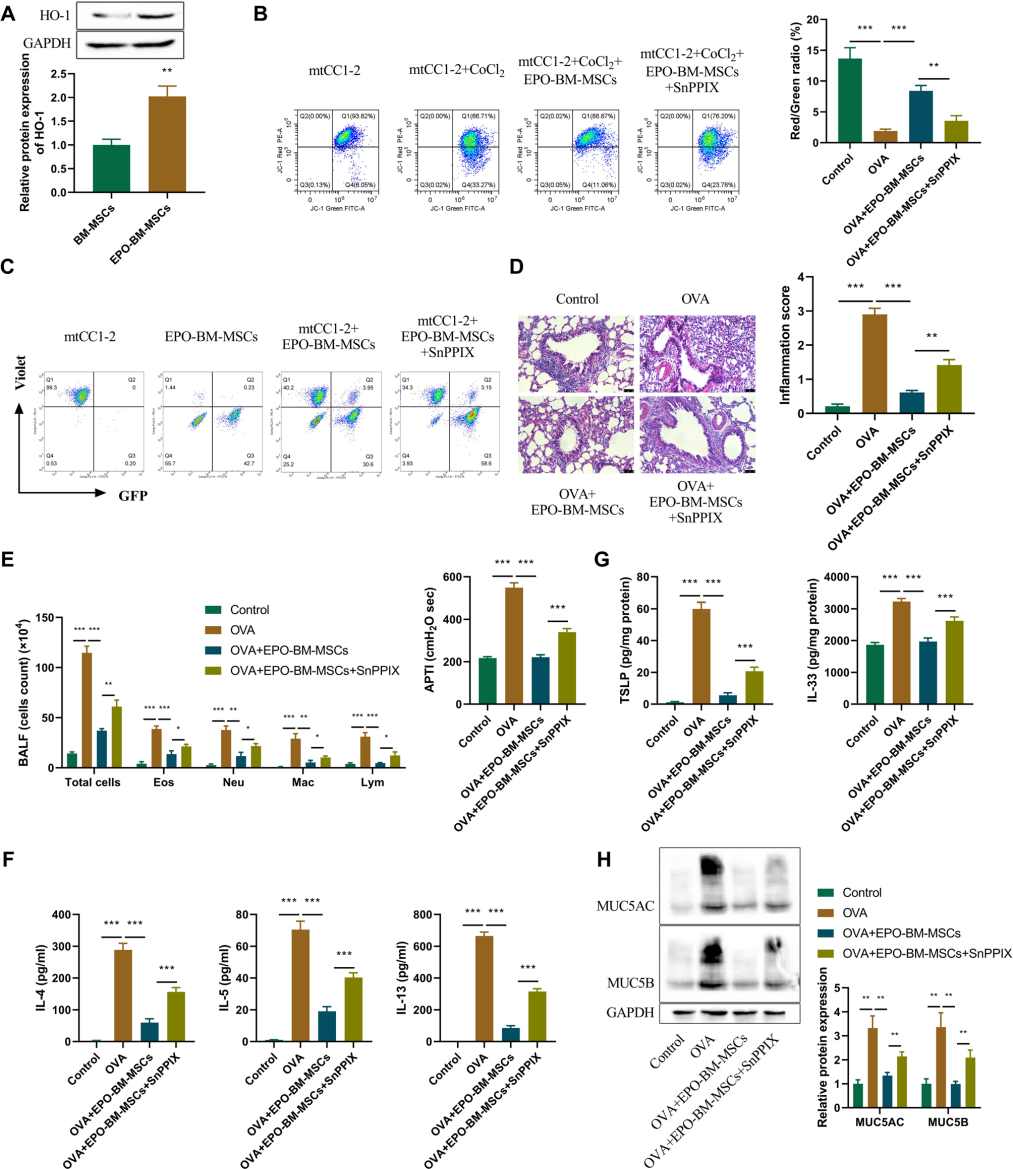
EPO-BM-MSCs-mediated upregulation of HO-1 promotes mitochondrial transfer from EPO-BM-MSCs to epithelial cells *in vitro* and enhances anti-inflammatory efficacy of EPO-BM-MSCs *in vivo*. **A** Immunoblotting of HO-1 protein in EPO-BM-MSCs and BM-MSCs. **B** mtCC1-2 cells were stimulated with or without CoCl_2_ for 12 h, or CoCl_2_-stimulated mtCC1-2 cells were co-cultured with EPO-BM-MSCs in normal media or media containing SnPPIX for 24 h. MMP of treated mtCC1-2 cells evaluated by flow cytometry. **C** mGFP-EPO-BM-MSCs (showing a green fluorescence) were co-cultured with mtCC1-2 cells labeled with CellTrace Violet (showing a blue fluorescence) in normal media or media containing SnPPIX. Flow cytometry analyzed the percentage of mtCC1-2 cells labeled with GFP and CellTrace Violet. (D-H) An OVA-induced mouse model of asthma was established, which was administrated with EPO-BM-MSCs or EPO-BM-MSCs + SnPPIX. At the end point, the BALF and lung tissues were obtained. Each group included six mice. **D** H&E staining of lung sections for histopathological observation. Scale bars: 50 μm. **E** Total cells, eosinophils (Eos), neutrophils (Neu), macrophages (Mac), and lymphocytes (Lym) were counted in the BALF. **F** Airway pressure-time index (APTI) was used to evaluate airway responsiveness. **G** ELISA for IL-4, IL-5, IL-13, TSLP and IL-33 secretion levels in the BALF. **H** Western blot analysis of MUC5AC and MUC5B expression in lung tissues treated with EPO-BM-MSCs ± SnPPIX. *P < 0.05, **P < 0.01, ***P < 0.001

Recent studies have highlighted the function of EPO in inducing HO-1 expression (El-Ashmawy et al. 2022; Sun et al. 2019), and numerous reports have proven the crucial role of HO-1 in mitochondrial function and activation (Shi et al. 2021; Yang et al. 2024a, 2024b). Intriguingly, in an attempt to elucidate the mechanism involved in mitochondrial transfer from EPO-BM-MSCs, we found that EPO-BM-MSCs exhibited higher protein levels of HO-1 than BM-MSCs (Fig.5A). To validate whether HO-1 is responsible for enhanced mitochondrial activation and transfer, SnPPIX, an inhibitor of HO-1, was used in vitro and in vivo. Flow cytometry analysis showed that SnPPIX treatment strongly reversed EPO-BM-MSCs-driven MMP restoration in CoCl_2_-stimulated mtCC1-2 cells (Fig. 5B). Similarly, after co-culture of mGFP-EPO-BM-MSCs with CellTrace Violet-labeled mtCC1-2 cells, SnPPIX treatment exerted a counteracting effect on EPO-BM-MSCs-mediated elevation in the percentage of GFP-positive mtCC1-2 cells (Fig. 5C), indicating that SnPPIX could partially abate mitochondrial transfer of EPO-BM-MSCs to mtCC1-2 cells. All these results suggest that the enhanced mitochondrial transfer of EPO-BM-MSCs to mtCC1-2 cells is dependent, at least in part, on HO-1 upregulation.

Moreover, in OVA-induced asthma mice, administration of SnPPIX remarkably counteracted EPO-BM-MSCs-imposed inhibition in lung inflammation (Fig. 5D), reduction in total cell count and inflammatory cell count in BALF (Fig. 5E), repression in airway responsiveness (Fig. 5F), as well as downregulation in IL-4, IL-5, IL-13, TSLP and IL-33 secretion levels in mouse BALF (Fig. 5G and H). Thus, HO-1 upregulation contributes to enhanced anti-inflammatory efficacy of EPO-BM-MSCs in OVA-induced asthma mice. Furthermore, Western blot analysis of lung tissues revealed that EPO-BM-MSCs markedly suppressed the expression of mucin markers MUC5AC and MUC5B in OVA-induced asthma mice (Fig. 5H). This inhibitory effect was reversed by co-administration of SnPPIX, indicating that HO-1 upregulation in EPO-BM-MSCs contributes to the reduction of mucus hypersecretion. These findings support the role of HO-1 in mediating both mitochondrial transfer and epithelial secretory function regulation.

### M-sec promotes TNT formation and Miro1 enhances mitochondrial transfer from EPO-BM-MSCs to mtCC1-2 cells

**Fig. 6:**
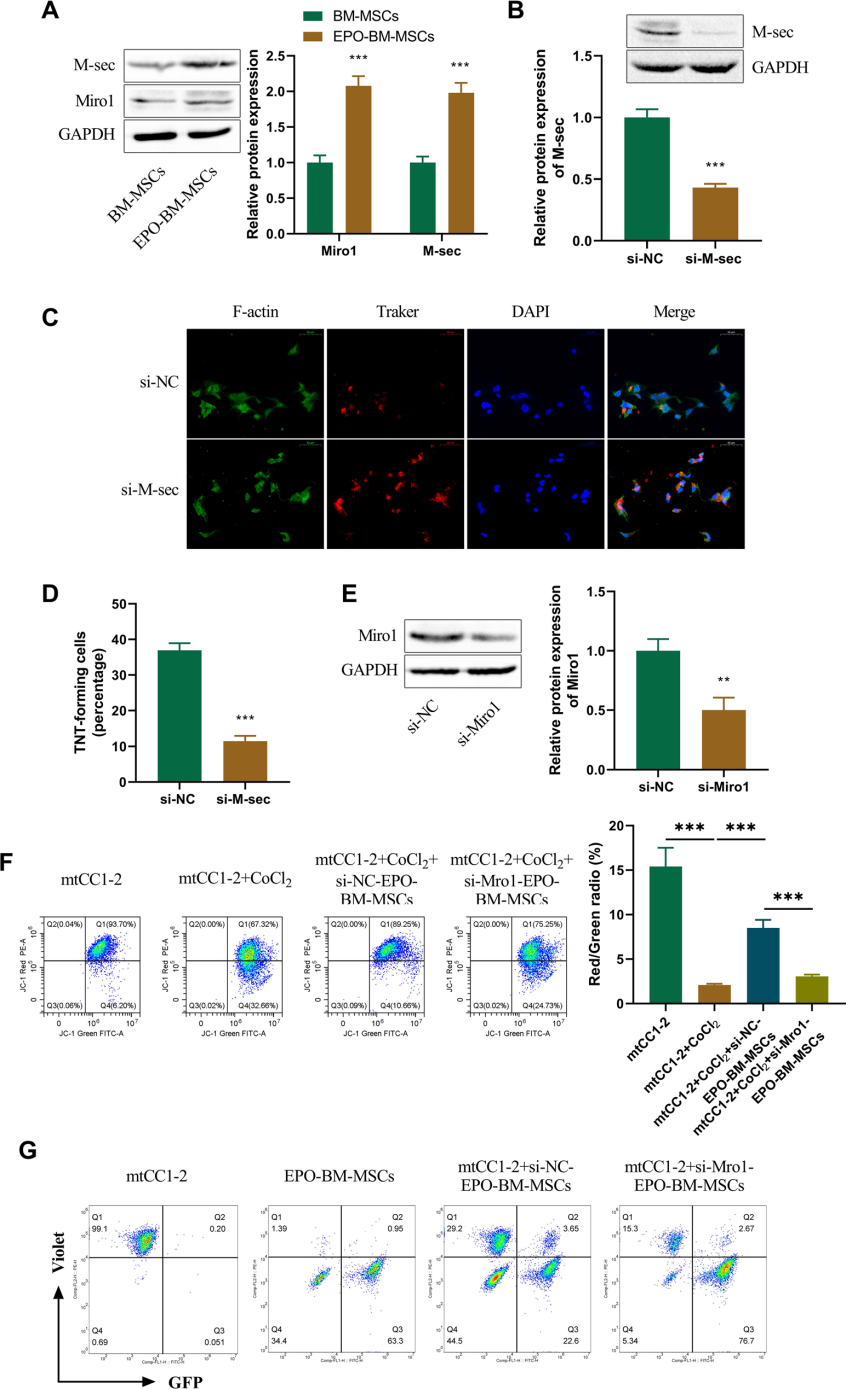
M-sec and Miro1 are involved in mitochondrial transfer from EPO-BM-MSCs to mtCC1-2 cells. **A** Relative expression of M-sec and Miro1 in EPO-BM-MSCs and BM-MSCs as examined by immunoblotting. **B** Immunoblotting of M-sec in si-NC- or si-M-sec-transfected EPO-BM-MSCs. **C** and **D** Fluorescence microscopy and the percentage of TNT-forming cells between si-NC-transfected EPO-BM-MSCs or si-M-sec-transfected EPO-BM-MSCs. **E** Immunoblotting of M-sec in si-NC- or si-Miro1-transfected EPO-BM-MSCs. **F** mtCC1-2 cells were stimulated with or without CoCl_2_ for 12 h, or CoCl_2_-stimulated mtCC1-2 cells were co-cultured with si-NC- or si-Miro1-transfected EPO-BM-MSCs. MMP of treated mtCC1-2 cells evaluated by flow cytometry. **G** Si-NC- or si-Miro1-transfected mGFP-EPO-BM-MSCs (showing a green fluorescence) were co-cultured with mtCC1-2 cells labeled with CellTrace Violet (showing a blue fluorescence). Flow cytometry analyzed the percentage of mtCC1-2 cells labeled with GFP and CellTrace Violet. **P < 0.01, ***P < 0.001

M-sec has been reported to contribute to TNT formation and thus induces mitochondrial horizontal transfer (Barutta et al. 2021). Growing evidence also suggests that Miro1 is essential for mitochondrial transfer from MSCs to epithelial cells (Tseng et al. 2021; Ahmad et al. 2014). We further elucidated whether the two molecules are involved in mitochondrial transfer from EPO-BM-MSCs to mtCC1-2 cells. Through immunoblot analysis, we observed that EPO-BM-MSCs had increased expression of M-sec and Miro1 compared with BM-MSCs (Fig.6A). To study the influence of M-sec on TNT formation between EPO-BM-MSCs, we used a specific M-sec-siRNA (si-M-sec) to silence its expression in EPO-BM-MSCs. The knockdown efficiency of si-M-sec was validated by immunoblotting (Fig. 6B). Depletion of M-sec strikingly suppressed TNT formation between EPO-BM-MSCs (Fig. 6C and D), suggesting that M-sec could promote TNT formation. To test the impact of Miro1 on mitochondrial activation and transfer, the Miro1-siRNA (si-Miro1) was used, which was verified to effectively deplete Miro1 expression in EPO-BM-MSCs (Fig. 6E). Miro1 knockdown in EPO-BM-MSCs strongly impaired MMP in CoCl_2_-stimulated mtCC1-2 cells (Fig. 6F). Moreover, reduced Miro1 expression suppressed mitochondrial transfer of EPO-BM-MSCs to mtCC1-2 cells, as presented by the decreased percentage of GFP-positive mtCC1-2 cells in the si-Miro1 group compared with the si-NC control (Fig. 6G). Collectively, these findings demonstrate the involvement of M-sec and Miro1 in mitochondrial transfer from EPO-BM-MSCs to mtCC1-2 cells.

## Discussion

Asthma, a chronic inflammatory disorder of the airways, is a common refractory disease (Agache et al. 2021). Emerging evidence has illustrated that BM-MSCs, with the ability of self-renewal and multi-directional differentiation, possess potential efficacy in treating asthma (Choi et al. 2022; Ou-Yang et al. 2011). Our previous study has established that EPO-BM-MSCs are capable of attenuating asthmatic airway inflammation and remodeling in OVA-challenged asthma mice (Han et al. 2018). In this study, we provide further evidence for the therapeutic efficacy of EPO-BM-MSCs in improving asthmatic phenotype in model mice. EPO-BM-MSCs can relieve lung inflammation and inflammatory cytokine production in OVA-induced mice (Han et al. 2018). Meantime, EPO-BM-MSCs exert a strong function in reducing collagen density, the thickness of smooth muscle layer, and the number of von Willebrand factor positive cells in mouse lungs (Han et al. 2018). In agreement with these observations, we have proven that intratracheal transplantation of EPO-BM-MSCs is more efficient for diminishing lung inflammation, airway reactivity, and Th2 inflammatory cytokine secretion than BM-MSCs in OVA-induced asthma mice.

Mitochondrial dysfunction of airway epithelial cells plays a pathogenic role in various pulmonary diseases including asthma (Zhao et al. 2023). Improving mitochondrial quality has been proposed as a promising strategy for disease treatment (Larson-Casey et al. 2020). Previous work has highlighted the rescue efficacy of MSCs in mitochondrial dysfunction (Dutra Silva et al. 2021). It’s worth noting that MSCs have been reported to ameliorate OVA-evoked epithelial mitochondrial dysfunction, thereby resulting in reduced airway inflammation in OVA asthma mice (Yao et al. 2018). Our results unveil the engraftment of EPO-BM-MSCs into the lungs after intratracheal transplantation in OVA-induced mice, which is consistent with the earlier study where pluripotent stem cell (iPSC)-derived MSCs by intratracheal administration can engraft into pulmonary epithelium in asthmatic mice (Yao et al. 2018). Utilizing in vivo and in vitro experiments, we demonstrate that EPO-BM-MSCs have the rescue efficacy to ameliorate epithelial mitochondrial dysfunction. Our results further demonstrate that EPO-BM-MSCs suppress the expression of mucin markers (MUC5AC and MUC5B) in asthmatic lungs, which is critical for alleviating airway obstruction—a hallmark of severe asthma. Mucus hypersecretion is driven by epithelial cell dysfunction and inflammatory signaling, and our data suggest that EPO-BM-MSCs restore epithelial homeostasis via mitochondrial transfer and HO-1-mediated anti-oxidative effects. The reduction in mucin expression complements the observed decrease in Th2 cytokines and airway inflammation, highlighting the multi-faceted therapeutic potential of EPO-BM-MSCs in asthma.

Numerous studies have highlighted the capacity of BM-MSCs for mitochondrial transfer to injured recipient cells via intercellular TNTs (Paliwal et al. 2018). This capacity endows BM-MSCs with therapeutic potential in various human disorders, such as acute lung injury, spinal cord injury, and dental pulp damage (Yao et al. 2023; Islam et al. 2012; Wang et al. 2023). Moreover, MSCs can donate mitochondria to rotenone-induced airway epithelial cells to relieve epithelial cell stress and injury (Ahmad et al. 2014). Through mitochondria donation, iPSC-derived MSCs diminish epithelial mitochondrial dysfunction to alleviate inflammation in OVA-challenged asthma mice (Yao et al. 2018). Our study shows the effective formation of TNTs between EPO-BM-MSCs and CoCl_2_-treated mtCC1-2 cells, which offers the possibility for mitochondrial transfer from EPO-BM-MSCs to mtCC1-2 cells. Importantly, we demonstrate, for the first time, that EPO-BM-MSCs can donate mitochondria to mtCC1-2 cells in vitro and pulmonary epithelial cells in vivo through intercellular TNTs.

HO-1, a pivotal anti-oxidant enzyme, plays a crucial role in mitochondrial function, activation and quality (Yang et al. 2024a, 2024b; Jiang et al. 2020). For example, in LPS-induced lung damage, HO-1 increase can affect the expression of mitochondrial mitophagy mediators (e.g. PINK1 and Parkin), maintain the dynamic process of mitochondrial fusion/fission, as well as contribute to enhanced mitochondrial biogenesis (Shi et al. 2019). In hepatocellular carcinoma cells, HO-1 depletion is found to be related to diminished MMP and mitochondrial structural damage (Yang et al. 2024a, 2024b). Moreover, HO-1 is involved in the protection of Prussian blue nanoparticles on mitochondria against oxidative stress-evoked injury (Xu et al. 2022). Importantly, HO-1 expressed in umbilical cord MSCs can help improve the ovarian function in a mouse model of premature ovarian failure (Yin et al. 2020)and alleviate neural inflammation and damage in stroke mice (Yang et al. 2024a, 2024b). HO-1-modified BM-MSCs exert an enhanced protective function in reducing ischemia/reperfusion injury by repressing ferroptosis (Wu et al. 2022). Furthermore, EPO has been reported to induce the nuclear translocation of Nrf2 and thus leads to increased HO-1 expression (El-Ashmawy et al. 2022; Sun et al. 2019). In this paper, we have found that HO-1 expression is increased in EPO-BM-MSCs compared with that in BM-MSCs. Importantly, we demonstrate that the enhanced mitochondrial transfer from EPO-BM-MSCs to epithelial cells, the increased mitochondrial activation of CoCl_2_-induced mtCC1-2 cells, and the enhanced anti-inflammatory efficacy of EPO-BM-MSCs in OVA-induced mice are dependent, at least in part, on HO-1 upregulation. In addition, our data show higher levels of M-sec, an inducer of TNT formation (Barutta et al. 2021), and Miro1, a well-established contributor in mitochondrial transfer from MSCs (Tseng et al. 2021), in EPO-BM-MSCs than BM-MSCs. M-sec overexpression can contribute to TNT formation by interacting with the Ral-exocyst pathway and thus mediates mitochondrial horizontal transfer (Barutta et al. 2021; Hase et al. 2009). Miro1 is a mitochondrial Rho-GTPase that has the ability to improve the metabolic benefit of mitochondrial transfer (Tseng et al. 2021). Increased expression of Miro1 in MSCs results in enhanced mitochondrial transfer to injured epithelial cells, promoting MSC rescue efficacy (Ahmad et al. 2014). We also prove the involvement of M-sec and Miro1 in mitochondrial transfer from EPO-BM-MSCs to mtCC1-2 epithelial cells. Mechanistically, HO-1 upregulation in EPO-BM-MSCs promotes TNT formation by enhancing M-sec expression (Fig.6A-B) and facilitates mitochondrial motility via Miro1, two critical steps for efficient mitochondrial transfer. HO-1 also protects mitochondrial integrity under oxidative stress, ensuring functional mitochondria are delivered to recipient cells. While our study focuses on HO-1-mediated TNT formation and Miro1-dependent mitochondrial transport, the EPO-Nrf2-HO-1 axis may also modulate broader mitochondrial quality control pathways (e.g., biogenesis, dynamics), potentially enhancing the efficiency of mitochondrial transfer. Future studies could explore interactions with other signaling modules, such as AMPK or EV-mediated pathways, to fully characterize the molecular network.

Recent advancements in the field of BM-MSCs therapy for asthma along with our current study have demonstrated promising results, positioning BM-MSCs and EPO-BM-MSCs as potential therapeutic options for asthma. In comparison to existing treatments, BM-MSC therapy offers several advantages. Traditional asthma treatments, such as bronchodilators and corticosteroids, primarily focus on symptom management rather than addressing the underlying inflammation and immune dysregulation. In contrast, BM-MSCs have the potential to target these root causes, providing a more holistic approach to asthma management. Furthermore, BM-MSC therapy may reduce reliance on long-term medications and their associated side effects. Despite these promising advantages, the use of BM-MSCs in asthma treatment also presents certain limitations. The mechanisms underlying the therapeutic effects of BM-MSCs remain incompletely understood, which hinders the optimization of treatment protocols. Additionally, the isolation, expansion, and differentiation of BM-MSCs require specialized techniques and facilities, limiting their widespread application. Moreover, the long-term safety and efficacy of BM-MSC therapy in asthma patients necessitate further evaluation in larger clinical trials. While the current study provides valuable insights into the potential of EPO-BM-MSC therapy for asthma, it is constrained by its sample size, type, and duration. Future research should prioritize conducting larger, multicenter trials to validate these findings and explore the optimal dosing and administration routes of EPO-BM-MSCs. Furthermore, studies are needed to elucidate the exact mechanisms of action of EPO-BM-MSCs in asthma, which could pave the way for the development of more targeted and effective therapies.

While our study identifies TNT-mediated mitochondrial transfer as a critical mechanism for EPO-BM-MSCs’ anti-inflammatory effects, the partial inhibition of these effects by Gap26 (a TNT disruptor) implies the involvement of additional pathways. Mesenchymal stem cells are known to exert paracrine effects through extracellular vesicles (EVs), including exosomes and microvesicles, which carry bioactive molecules such as miRNAs, cytokines (e.g., IL-10, TGF-β), and proteins that modulate immune responses and epithelial cell function (Kou et al. 2022). For example, MSC-derived EVs have been shown to reduce inflammation by inhibiting Th2 cytokine production and promoting regulatory T cell activation in asthma models (Kim and Cho 2022). Although our experiments did not directly investigate EVs, it is plausible that EPO-BM-MSCs may simultaneously utilize EVs to deliver anti-inflammatory cargo, complementing TNT-dependent mitochondrial transfer. The limited efficacy of Gap26 could thus reflect the combined contributions of both TNT-mediated mitochondrial donation and EV-mediated signaling, highlighting the complex intercellular communication networks employed by EPO-BM-MSCs. Future studies are warranted to characterize the specific EV cargo and functional roles in asthma inflammation, further dissecting the multi-modal therapeutic actions of these modified MSCs.

While our study uses mouse bone marrow MSCs (BM-MSCs) to investigate the therapeutic mechanisms of EPO modification, we recognize that clinical translation would require transitioning to human-derived MSCs (hMSCs) to address regulatory and ethical considerations. Mouse MSCs serve as a valuable preclinical model for mechanistic exploration, but human applications necessitate compliance with Good Manufacturing Practice (GMP) standards for cell production, ensuring safety, purity, and efficacy. Key future steps include: ① Evaluating the tumorigenicity, immune compatibility, and long-term effects of hMSCs in preclinical models, as regulatory bodies (e.g., FDA, EMA) require extensive safety data before approving stem cell therapies. ② Ensuring hMSCs are sourced ethically (e.g., from adult tissues or induced pluripotent stem cells) to mitigate concerns about embryonic stem cell use. ③ Designing phase I/II trials to assess the safety and preliminary efficacy of EPO-hMSCs in asthma patients, with strict ethical oversight and regulatory compliance.

While our study demonstrates the short-term therapeutic effects of EPO-BM-MSCs in asthma models, the long-term impacts of this treatment remain unaddressed and warrant further investigation. Key areas for future research include: ① Evaluating potential risks such as tumorigenicity, immune sensitization, or unintended tissue remodeling following repeated or prolonged EPO-BM-MSC administration in preclinical models. ② Assessing whether the observed anti-inflammatory and mitochondrial protective effects persist over time, including the durability of mitochondrial transfer and HO-1 signaling in epithelial cells. ③ Designing preclinical studies with extended follow-up periods to inform dose regimens and treatment intervals for future clinical trials, ensuring both efficacy and safety in patients. These limitations highlight that while our findings establish a mechanistic basis for EPO-BM-MSCs in asthma, comprehensive long-term studies are essential to validate their therapeutic potential and address regulatory requirements for clinical use.

**Fig. 7:**
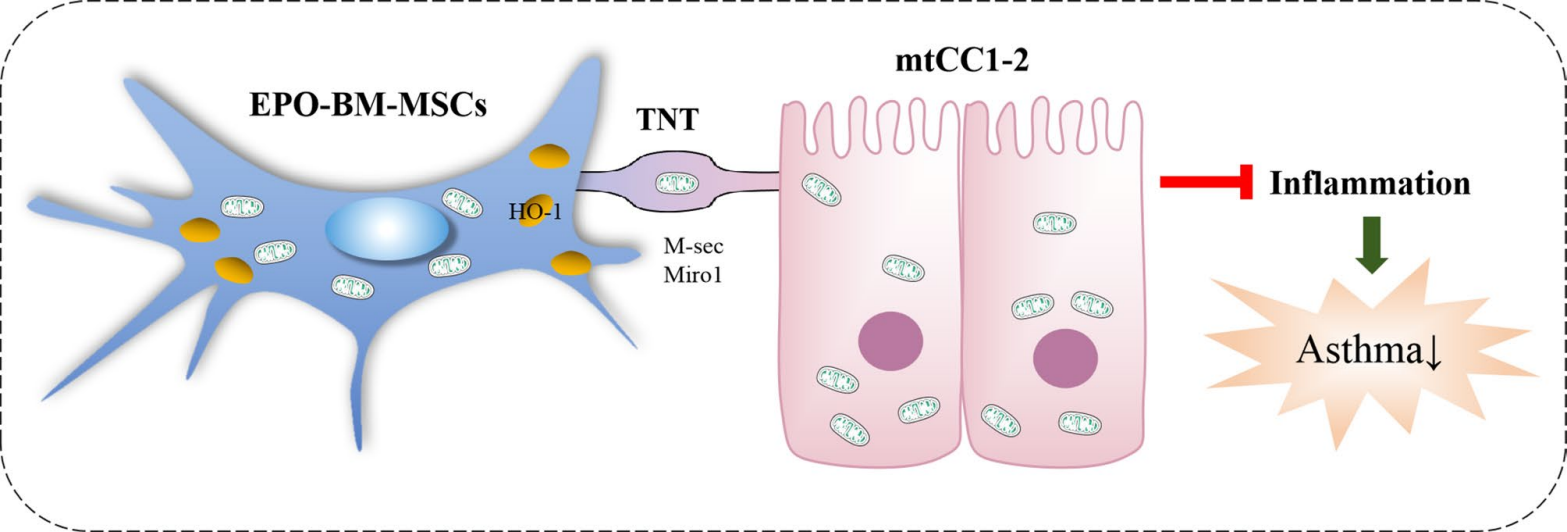
Schematic of the mechanism of EPO-BM-MSCs’s therapeutic efficacy on asthma. EPO-BM-MSCs upregulate HO-1, M-sec, and Miro1 expression to enhance mitochondrial donation to epithelial cells by intercellular TNTs, thereby alleviating airway inflammation in asthma

Collectively, we have demonstrated the therapeutic efficacy of EPO-BM-MSCs, with the ability to rescue epithelial cell injury by mitochondrial donation by upregulating HO-1, M-sec, and Miro1, in asthma inflammation (Fig. 7). Our study provides novel evidence for the protective efficacy of EPO-BM-MSCs in alleviating asthmatic phenotype.

## Data Availability

The data that are available from the corresponding author upon reasonable request.

## References

[CR1] Agache I, Eguiluz-Gracia I, Cojanu C, Laculiceanu A, Del Giacco S, Zemelka-Wiacek M, et al. Advances and highlights in asthma in 2021. Allergy. 2021;76(11):3390–407. 34392546 10.1111/all.15054

[CR2] Ahmad T, Mukherjee S, Pattnaik B, Kumar M, Singh S, Kumar M, et al. Miro1 regulates intercellular mitochondrial transport & enhances mesenchymal stem cell rescue efficacy. EMBO J. 2014;33(9):994–1010. 24431222 10.1002/embj.201386030 PMC4193933

[CR3] Arshad M, Jalil F, Jaleel H, Ghafoor F. Bone marrow derived mesenchymal stem cells therapy for rheumatoid arthritis - a concise review of past ten years. Mol Biol Rep. 2023;50(5):4619–29. 36929285 10.1007/s11033-023-08277-9

[CR4] Barutta F, Kimura S, Hase K, Bellini S, Corbetta B, Corbelli A, et al. Protective role of the M-Sec-Tunneling nanotube system in podocytes. J Am Soc Nephrol. 2021;32(5):1114–30. 33722931 10.1681/ASN.2020071076 PMC8259684

[CR5] Borcherding N, Brestoff JR. The power and potential of mitochondria transfer. Nature. 2023;623(7986):283–91. 37938702 10.1038/s41586-023-06537-z PMC11590279

[CR6] Choi JY, Hur J, Jeon S, Jung CK, Rhee CK. Effects of human adipose tissue- and bone marrow-derived mesenchymal stem cells on airway inflammation and remodeling in a murine model of chronic asthma. Sci Rep. 2022;12(1):12032. 35835804 10.1038/s41598-022-16165-8 PMC9283392

[CR7] Dhlamini Q, Wang W, Feng G, Chen A, Chong L, Li X, et al. FGF1 alleviates LPS-induced acute lung injury via suppression of inflammation and oxidative stress. Mol Med. 2022;28(1):73. 35764933 10.1186/s10020-022-00502-8 PMC9238076

[CR8] Dutra Silva J, Su Y, Calfee CS, Delucchi KL, Weiss D, McAuley DF, et al. Mesenchymal stromal cell extracellular vesicles rescue mitochondrial dysfunction and improve barrier integrity in clinically relevant models of ARDS. Eur Respir J. 2021;58(1):2002978. 33334945 10.1183/13993003.02978-2020 PMC8318599

[CR9] El-Ashmawy NE, Al-Ashmawy GM, Farag AA, Ibrahim AO. Hemin versus erythropoietin: possible role in Nrf2/HO-1 signaling pathway in rats with nephrotoxicity. Biomed Pharmacother. 2022;156:113971. 36411647 10.1016/j.biopha.2022.113971

[CR10] Han XP, Zhang FQ, Tan XS, Liu L, Ma WX, Ou-Yang HF, et al. EPO modified MSCs can inhibit asthmatic airway remodeling in an animal model. J Cell Biochem. 2018;119(1):1008–16. 28686347 10.1002/jcb.26268

[CR11] Hase K, Kimura S, Takatsu H, Ohmae M, Kawano S, Kitamura H, et al. M-sec promotes membrane nanotube formation by interacting with Ral and the exocyst complex. Nat Cell Biol. 2009;11(12):1427–32. 19935652 10.1038/ncb1990

[CR12] Islam MN, Das SR, Emin MT, Wei M, Sun L, Westphalen K, et al. Mitochondrial transfer from bone-marrow-derived stromal cells to pulmonary alveoli protects against acute lung injury. Nat Med. 2012;18(5):759–65. 22504485 10.1038/nm.2736 PMC3727429

[CR13] Izadi M, Sadr Hashemi Nejad A, Moazenchi M, Masoumi S, Rabbani A, Kompani F, et al. Mesenchymal stem cell transplantation in newly diagnosed type-1 diabetes patients: a phase I/II randomized placebo-controlled clinical trial. Stem Cell Res Ther. 2022;13(1):264. 35725652 10.1186/s13287-022-02941-w PMC9208234

[CR14] Jiang N, Zhao H, Han Y, Li L, Xiong S, Zeng L, et al. HIF-1α ameliorates tubular injury in diabetic nephropathy via HO-1-mediated control of mitochondrial dynamics. Cell Prolif. 2020;53(11):e12909. 32975326 10.1111/cpr.12909 PMC7653251

[CR15] Kim SD, Cho KS. Immunomodulatory effects of mesenchymal stem Cell-Derived extracellular vesicles in allergic airway disease. Life (Basel). 2022;12(12):1994. 36556359 10.3390/life12121994 PMC9786036

[CR16] Kou M, Huang L, Yang J, Chiang Z, Chen S, Liu J, et al. Mesenchymal stem cell-derived extracellular vesicles for immunomodulation and regeneration: a next generation therapeutic tool? Cell Death Dis. 2022;13(7):580. 35787632 10.1038/s41419-022-05034-x PMC9252569

[CR17] Larson-Casey JL, He C, Carter AB. Mitochondrial quality control in pulmonary fibrosis. Redox Biol. 2020;33:101426. 31928788 10.1016/j.redox.2020.101426 PMC7251238

[CR18] Lin RZ, Im GB, Luo AC, Zhu Y, Hong X, Neumeyer J, et al. Mitochondrial transfer mediates endothelial cell engraftment through mitophagy. Nature. 2024;629(8012):660–8. 38693258 10.1038/s41586-024-07340-0 PMC11574736

[CR19] Liu Y, Fu T, Li G, Li B, Luo G, Li N, et al. Mitochondrial transfer between cell crosstalk - an emerging role in mitochondrial quality control. Ageing Res Rev. 2023;91:102038. 37625463 10.1016/j.arr.2023.102038

[CR20] Miller RL, Grayson MH, Strothman K. Advances in asthma: new understandings of asthma’s natural history, risk factors, underlying mechanisms, and clinical management. J Allergy Clin Immunol. 2021;148(6):1430–41. 34655640 10.1016/j.jaci.2021.10.001

[CR21] Ou-Yang HF, Huang Y, Hu XB, Wu CG. Suppression of allergic airway inflammation in a mouse model of asthma by exogenous mesenchymal stem cells. Exp Biol Med (Maywood). 2011;236(12):1461–7. 22114062 10.1258/ebm.2011.011221

[CR22] Paliwal S, Chaudhuri R, Agrawal A, Mohanty S. Regenerative abilities of mesenchymal stem cells through mitochondrial transfer. J Biomed Sci. 2018;25(1):31. 29602309 10.1186/s12929-018-0429-1 PMC5877369

[CR23] Purwaningrum M, Jamilah NS, Purbantoro SD, Sawangmake C, Nantavisai S. Comparative characteristic study from bone marrow-derived mesenchymal stem cells. J Vet Sci. 2021;22(6):e74. 34697921 10.4142/jvs.2021.22.e74 PMC8636658

[CR24] Sadanandan J, Sathyanesan M, Liu Y, Tiwari NK, Newton SS. Carbamoylated erythropoietin-induced cerebral blood perfusion and vascular gene regulation. Int J Mol Sci. 2023;24(14):11507. 37511274 10.3390/ijms241411507 PMC10380798

[CR25] Sagar S, Faizan MI, Chaudhary N, Singh V, Singh P, Gheware A, et al. Obesity impairs cardiolipin-dependent mitophagy and therapeutic intercellular mitochondrial transfer ability of mesenchymal stem cells. Cell Death Dis. 2023;14(5):324. 37173333 10.1038/s41419-023-05810-3 PMC10181927

[CR26] Scholz GA, Leichtle AB, Scherer A, Arndt U, Fiedler M, Aeberli D, et al. The links of hepcidin and erythropoietin in the interplay of inflammation and iron deficiency in a large observational study of rheumatoid arthritis. Br J Haematol. 2019;186(1):101–12. 30941747 10.1111/bjh.15895

[CR27] Shi J, Yu J, Zhang Y, Wu L, Dong S, Wu L, et al. PI3K/Akt pathway-mediated HO-1 induction regulates mitochondrial quality control and attenuates endotoxin-induced acute lung injury. Lab Invest. 2019;99(12):1795–809. 31570770 10.1038/s41374-019-0286-x

[CR28] Sinclair KA, Yerkovich ST, Hopkins PM, Chambers DC. Characterization of intercellular communication and mitochondrial donation by mesenchymal stromal cells derived from the human lung. Stem Cell Res Ther. 2016;7(1):91. 27406134 10.1186/s13287-016-0354-8 PMC4942965

[CR29] Shi J, Yu T, Song K, Du S, He S, Hu X, et al. Dexmedetomidine ameliorates endotoxin-induced acute lung injury in vivo and in vitro by preserving mitochondrial dynamic equilibrium through the HIF-1a/HO-1 signaling pathway. Redox Biol. 2021;41:101954. 33774474 10.1016/j.redox.2021.101954 PMC8027777

[CR30] Sun C, Yao Y, Zhang C, Tong D, Xie B. EPO attenuates Cisplatin-induced ototoxicity in HEI-OC1 auditory cell via the Nrf2-ARE signaling pathway. Otol Neurotol. 2019;40(7):965–71. 31135681 10.1097/MAO.0000000000002288

[CR31] Tseng N, Lambie SC, Huynh CQ, Sanford B, Patel M, Herson PS, et al. Mitochondrial transfer from mesenchymal stem cells improves neuronal metabolism after oxidant injury in vitro: the role of Miro1. J Cereb Blood Flow Metab. 2021;41(4):761–70. 32501156 10.1177/0271678X20928147 PMC7983509

[CR32] Wang K, Zhou L, Mao H, Liu J, Chen Z, Zhang L. Intercellular mitochondrial transfer alleviates pyroptosis in dental pulp damage. Cell Prolif. 2023;56(9):e13442. 37086012 10.1111/cpr.13442 PMC10472516

[CR33] Wu L, Tian X, Zuo H, Zheng W, Li X, Yuan M, et al. MiR-124-3p delivered by exosomes from Heme oxygenase-1 modified bone marrow mesenchymal stem cells inhibits ferroptosis to attenuate ischemia-reperfusion injury in steatotic grafts. J Nanobiotechnol. 2022;20(1):196. 10.1186/s12951-022-01407-8 PMC9026664 35459211

[CR34] Xu Z, Liu Y, Ma R, Chen J, Qiu J, Du S, et al. Thermosensitive hydrogel incorporating Prussian blue nanoparticles promotes diabetic wound healing via ROS scavenging and mitochondrial function restoration. ACS Appl Mater Interfaces. 2022;14(12):14059–71. 35298140 10.1021/acsami.1c24569

[CR35] Yang Y, Liu Q, Deng S, Shao Q, Peng L, Ling Y, et al. Human umbilical cord derived mesenchymal stem cells overexpressing HO-1 attenuate neural injury and enhance functional recovery by inhibiting inflammation in stroke mice. CNS Neurosci Ther. 2024b;30(2):e14412. 37592866 10.1111/cns.14412 PMC10848045

[CR36] Yang R, Gao W, Wang Z, Jian H, Peng L, Yu X, et al. Polyphyllin I induced ferroptosis to suppress the progression of hepatocellular carcinoma through activation of the mitochondrial dysfunction via Nrf2/HO-1/GPX4 axis. Phytomedicine. 2024a;122:155135. 37856990 10.1016/j.phymed.2023.155135

[CR37] Yao S, Pang M, Wang Y, Wang X, Lin Y, Lv Y, et al. Mesenchymal stem cell attenuates spinal cord injury by inhibiting mitochondrial quality control-associated neuronal ferroptosis. Redox Biol. 2023;67:102871. 37699320 10.1016/j.redox.2023.102871 PMC10506061

[CR38] Yao Y, Fan XL, Jiang D, Zhang Y, Li X, Xu ZB, et al. Connexin 43-mediated mitochondrial transfer of iPSC-MSCs alleviates asthma inflammation. Stem Cell Reports. 2018;11(5):1120–35. 30344008 10.1016/j.stemcr.2018.09.012 PMC6234920

[CR39] Yin N, Wu C, Qiu J, Zhang Y, Bo L, Xu Y, et al. Protective properties of heme oxygenase-1 expressed in umbilical cord mesenchymal stem cells help restore the ovarian function of premature ovarian failure mice through activating the JNK/Bcl-2 signal pathway-regulated autophagy and upregulating the circulating of CD8(+)CD28(-) T cells. Stem Cell Res Ther. 2020;11(1):49. 32019599 10.1186/s13287-019-1537-x PMC7001243

[CR40] Zhao L, Gao J, Chen G, Huang C, Kong W, Feng Y, et al. Mitochondria dysfunction in airway epithelial cells is associated with type 2-low asthma. Front Genet. 2023;14:1186317. 37152983 10.3389/fgene.2023.1186317 PMC10160377

